# Measurement of Oxidative Stress Index (OSI) in Penile Corpora Cavernosa and Peripheral Blood of Peyronie’s Disease Patients: A Report of 49 Cases

**DOI:** 10.3390/metabo14010055

**Published:** 2024-01-15

**Authors:** Gianni Paulis, Andrea Paulis, Giovanni De Giorgio, Salvatore Quattrocchi

**Affiliations:** 1 Peyronie’s Care Center, Department of Urology and Andrology, Castelfidardo Clinical Analysis Center, 00185 Rome, Italy; 2 Neurosystem for Applied Psychology and Neuroscience, Janet Clinical Centre, 00195 Rome, Italy; andrea.fx.94@gmail.com; 3 Section of Ultrasound Diagnostics, Department of Urology and Andrology, Castelfidardo Clinical Analysis Center, 00185 Rome, Italy; g.degiorgio@analisiclinichecastelfidardo.it; 4 Clinical Analysis Laboratory, Castelfidardo Clinical Analysis Center, 00185 Rome, Italy; salvatore.quattrocchi2@gmail.com

**Keywords:** Peyronies’s disease, oxidative stress, oxidative stress index, reactive oxygen species (ROS), total oxidant status, total antioxidant status, antioxidants, anxiety, depression

## Abstract

Peyronie’s disease (PD) is a chronic inflammatory disease affecting the penile albuginea. Oxidative stress (OS) is important for the development of the disease; therefore, it seemed interesting to us to directly measure OS at both the site of the disease and in peripheral blood. For a precise OS study, it is necessary to evaluate not only the single results of the total oxidant status (TOS) and total antioxidant status (TAS) but also their ratio: OS index (OSI) (arbitrary unit) = TOS/TAS × 100. This study included 49 PD patients examined and diagnosed in our Peyronie’s care center and a control group of 50 cases. We collected blood samples from both the penis and a vein in the upper extremity; we used d-ROMs and PAT-test (FRAS kit) for OS measurement. Pearson’s study found a statistical correlation between penile OSI values and PD plaque volumes: *p*-value = 0.002. No correlation was found between systemic OSI values and PD plaque volumes: *p*-value = 0.27. Penile OSI values were significantly reduced after the elimination of the PD plaque (*p* < 0.00001). The mean value of the penile OSI indices in the PD patients after plaque elimination corresponded to 0.090 ± 0.016 (*p* = 0.004). The comparison between the penile OSI values of the PD patients (with plaque elimination) and the control group revealed no statistically significant differences (*p* = 0.130). The absence of a correlation between Peyronie’s plaque volume and systemic OSI values indicates that it is preferable to carry out the OS study by taking a sample directly from the site of the disease. By carrying out a penile OSI study, it would be possible to obtain a precise plaque-volume-dependent oxidative marker. Even if the study did not demonstrate any correlation between OSI indices and anxious–depressive state, we detected a high prevalence of anxiety (81.6%) and depression (59.1%) in PD patients.

## 1. Introduction

Peyronie’s disease (PD) is a disease that consists of chronic inflammation located in the tunica albuginea of the penile corpora cavernosa. There is a genetic predisposition for this disease with dominant autonomic inheritance [1,2]. In the literature, the prevalence of PD varies from 3.2 to 13% [3,4,5,6]. This disease progressively evolves into a fibrous area (plaque) that focally reduces the elasticity of the penis, causing the following symptoms: deformation (curving and/or shortening, depression, incision, hourglass appearance), pain, erectile dysfunction (ED), and anxious–depressive state [7,8,9,10]. Although the pathogenesis has not yet been completely clarified, the most accredited etiopathogenetic theory is that of local trauma [11,12,13,14]. Following penile trauma, a blood collection would form that, instead of being reabsorbed, would attract inflammatory cells and large quantities of fibrogenic factors, cytokines, and free radicals (oxidative stress) with the secondary local hyperproduction of collagen (plaque) [15,16,17,18,19,20,21,22]. PD presents in a unifocal manner (single plaque) in the majority of cases (78–84%) but can also occur in multiple areas of the penile corpora cavernosa [9]. Over the last two decades, oxidative stress (OS) has proven to be very important and crucial for plaque formation and the evolution of the disease itself [15,16,17,18,19,20,21,22,23]. The disease presents and evolves in two phases. The first (active phase of inflammation) lasts approximately 12–18 months and coincides with the formation and growth of the plaque. It is in this phase that conservative treatment is indicated: oral substances (vitamin E, colchicine, potaba, tamoxifen, pentoxifylline/PTX and other antioxidants, nonsteroidal anti-inflammatory drugs/NSAIDs, phosphodiesterase 5/PDE-5 inhibitors); penile infiltrations with antifibrogenic substances, such as verapamil, corticosteroids, interferon-α2b, pentoxifylline/PTX, hyaluronic acid, and clostridium histolyticum collagenase/CCH; and physical therapies, such as extracorporeal shock wave therapy/ESWT, iontophoresis, penile traction devices, and vacuum devices. The second phase of PD consists of the stabilization of PD with the consequent stabilization of the deformation and the disappearance of pain. This is the phase of the disease where surgical intervention is indicated (corporoplasty, plaque incision, and/or implantation of penile prosthesis) [7,19,24,25,26,27,28,29,30,31,32,33,34,35,36,37,38,39,40,41,42,43,44,45,46]. Peyronie’s fibrous plaque can calcify in approximately 20–36% of cases [47,48].

Since OS plays an important role in PD, it is our opinion that, to obtain a more precise evaluation of OS in the disease under consideration, it is more appropriate to evaluate the oxidative state in both the penile corpora cavernosa and peripheral blood. There are already studies in the literature that have evaluated OSI in secretions (tears, saliva) taken from disease sites (ocular rosacea, periodontitis) [49,50].

This article is the first study in which OS is evaluated by calculating the OS index in blood samples taken directly from the corpora cavernosa of the penis of patients suffering from PD. The study of OS and OSI is also useful, above all, to evaluate the effectiveness of treatments for different pathologies, and in our case, it was very useful to evaluate the effectiveness of antioxidant therapy in patients suffering from PD. Until some time ago, our evaluation was based solely on ultrasound investigation, which allowed for us to examine the progressive reduction in PD plaque until its elimination.

With this new approach, which involves the study of OS in the penile corpora cavernosa, it would be possible to precisely detect the “chemical” evidence of the regression of the disease in the site where it was previously present. Until now, we could only use the ultrasound image as documentation of the disappearance of PD plaque.

## 2. Materials and Methods

### 2.1. Study Population

This study included 49 patients found to be affected by PD; these patients received the diagnosis and were examined in our Peyronie’s Care Center between June 2017 and January 2023. This study included a control group composed of 50 cases of healthy subjects without PD or other acute or chronic organic pathologies. This study was conducted in compliance with the principles contained in the Declaration of Helsinki (Fortaleza, 2013): all study subjects were contacted and provided their informed consent to the study. The sensitive data were, however, anonymized in compliance with privacy according to LEGISLATIVE DECREE 10 August 2018, n. 101 adapted to the GDPR.

The authors conducted this study on OS by collecting and analyzing the existing data from all patients, including those in the control group, from their medical records archived at our andrology clinic.

We specifically focused on the analysis of data related to the study of OS in PD patients before starting treatment with antioxidants and also after treatment. However, these treatments are not the subject of this article.

### 2.2. Purpose of This Study

OS can be measured by evaluating blood levels derived from the reactive oxygen metabolites (ROMs) (total oxidant status/TOS) and blood levels of total antioxidant status (TAS). To determine these values, the tests we used were the d-ROMs test and the plasma antioxidant test (PAT); concentrations of hydrogen peroxide (d-ROMs) and vitamin C (PAT) are used as reference standards for oxidative metabolites and antioxidant capacity (AC), respectively [51,52].

For a precise evaluation of oxidative stress (OS), it is necessary to evaluate both the single results of d-ROMs and PAT and their ratio: oxidative stress index (arbitrary unit) = d-ROMs/PAT × 100. The OS index (OSI), initially proposed in 2003 (Erel et al.), increases proportionally to any imbalance in the oxidative state [53,54,55,56].

There are numerous kits on the market to evaluate OS in blood samples; more specifically, we used a machine (FRAS 5) with related available kits [57]. With this device, it is possible to determine the redox balance in the sample using the d-ROMs Fast (measurement of free radicals) and PAT (measurement of antioxidant power) tests and, therefore, to calculate the OSI. In most cases, the study of OS in various diseases is conducted on blood samples taken from a peripheral vein; however, in this way, only the “general” oxidative state of the patient is analyzed [53,54,55,56,58,59,60,61,62].

The main aim of this study was to measure free radicals (d-ROM) and antioxidant capacity (PAT) in the peripheral blood and penile corpora cavernosa of patients with PD in order to measure OS directly at the site of the disease. With this study, we want to demonstrate whether OSI is reduced after treatment with antioxidants. This study includes both PD patients still undergoing treatment (24 cases) and PD patients who have already achieved ultrasound evidence of the disappearance of the PD plaque (25 cases).

To specify the actual state of the OS, we calculated the OS index using the relative formula.

Other aims of this study were as follows:

- Evaluation of the OS index in healthy subjects without PD or other acute or chronic organic pathologies;
- The search for a possible relationship between the value of the OS index detected in the penile corpora cavernosa (penile OSI value) and the volume of the disease area (plaque);
- The search for a possible relationship between the values of the systemic OSI (blood sampling from a peripheral vein) and the volumes of the disease area (plaque);
- The search for a possible relationship between the values of systemic OSI and penile OSI;
- The identification of penile OSI values indicative of regression of the disease area (PD plaque);
- The search for a possible relationship between values of systemic OSI and ongoing chronic pathology;
- The search for a possible relationship between penile OSI values and ongoing chronic pathology;
- The search for a possible relationship between penile OSI values and an anxious–depressive state (and its prevalence);
- The search for a possible relationship between systemic OSI values and an anxious–depressive state (and its prevalence).

#### 2.2.1. Inclusion and Exclusion Criteria

*Inclusion criteria* were patients affected by PD who were between 21 and 70 years of age and had available data that reported the results of a thorough clinical history examination (including all diseases); and the diagnosis of PD at the conclusion of the following assessments: performing penile palpation, photographic documentation of the penile deformation (according to Kelâmi) with a goniometric measurement of the angulation, penile eco-color Doppler ultrasound with plaque measurements (in the three dimensions, in mm) and volume calculation (mm^3^) using the ellipsoid formula (volume = 0.524 × length × width × thickness), and completion of the International Index of Erectile Function (IIEF) questionnaire for erectile function measurement, the Patient Health Questionnaire-9 (PHQ-9, concerning depression), the Generalized Anxiety Disorder Questionnaire-7 (GAD-7, concerning anxiety), and the visual analogue scale (VAS) questionnaire for measuring pain [63,64,65,66,67,68,69].

All patients (including the subjects of the control group) must have performed the OS study (d-ROMs and PAT) with subsequent calculation of the OSI with blood samples directly from the penile corpora cavernosa with a 25 G needle and from the peripheral blood (standard sampling from a vein of the upper extremity). Before the OS study, the patient must have fasted since the previous evening.

Each patient with PD must have performed the OS study before and after their antioxidant treatment (with a time interval of at least 6 months); the subsequent check-up after treatment must have been carried out more than once (at least one check-up) and always at least 6 months apart during the course of treatment.

During subsequent checks, each patient must have temporarily interrupted the antioxidant treatment for at least 10 days prior to the blood tests.

*Exclusion criteria* were PD patients with the following pathological situations (ongoing or recent within the last three months): infection (viral, bacterial, or other), acute inflammatory disease (gout attack, tooth abscess, burn, hemorrhoidal crisis, thrombophlebitis, prostatitis, acute dermatitis, allergic episode, etc.), thyroid hyperfunction, obesity, hypertensive crisis, ischemic and/or infarct episode, diabetes mellitus, liver dysfunction, renal failure, neoplastic disease, surgery, periodontal dental treatment, and, in any case, all conditions that cause a disturbance of the physiological redox balance (OS).

A total of 49 patients who had undergone an OS study and met the inclusion criteria were extracted from the total group of PD patients treated at our care center and included in this study.

Of these 49 patients (aged between 27 and 68 years), 25 of them had already been successfully treated with the disease being resolved, whilst the other 24 patients had not yet completed the entire treatment cycle.

All PD patients underwent an OS examination before and after treatment, at least 6 months after therapy. The treatment to which the PD patients were subjected was as follows: oral L-carnitine 1000 mg + bilberry 180 mg + propolis 700 mg + ginkgo biloba 240 mg + silymarin 400 mg + coenzyme Q-10 100 mg + vitamin C 50 mg + vitamin E 48 mg + superoxide dismutase 11,000 IU/g 10 mg/daily and topical diclofenac gel 4%/2 times daily + peri-plaque penile injections (only in the case of plaques with volume ≥ 100 mm^3^): pentoxifylline 100 mg (30 G needle) every month for 12 months and, then, 1 penile injection every 2 months for 12 months (18 total injections).

Given the difficulty in finding “normal” cases to include in the control group, we included 50 cases of subjects not affected by PD who had undergone OS studies (both of the peripheral blood and the penile corpora cavernosa) before carrying out a penile dynamic color Doppler ultrasound for suspected ED in the same session. In all 50 cases, the results of their respective dynamic ultrasound studies had shown normal results with the final diagnosis of exclusively psychogenic ED (note that their hormone tests were also normal).

We excluded from the present study all cases where a vascular or hormonal problem was found to be the cause of ED; furthermore, all patients who fell within the previously listed exclusion criteria were excluded.

#### 2.2.2. Data Collection

We collected all the demographic data of the PD patients and the characteristics of the ultrasound examination (location, size, and volume of the plaque), the type of deformation and the degree of penile curvature (if present), the degree of ED possibly present, the degree of any associated anxious–depressive state, and the degree of pain possibly present.

We also recorded all the demographic and clinical data relating to the 50 cases of the control group.

#### 2.2.3. Sample Collection

Blood samples for the measurement of OS were collected from both the penile corpora cavernosa and a vein of the upper extremity. The sampling from the penile corpora cavernosa (measurement of local OS) was carried out with a syringe and 25 G needle, avoiding puncturing the PD plaque. The amount of blood collected from the penile corpora cavernosa was 0.5 mL (500 µL). The sampling from the peripheral vein of the upper extremity, necessary for the evaluation of systemic OS, was carried out with a standard method; the quantity of blood aspirated was approximately 1–2 mL in order to ensure at least 0.5 mL (500 µL) for use for the exam. As soon as they were collected, the blood samples were immediately poured into a heparinized (and not EDTA) tube.

#### 2.2.4. Plasma Collection

The samples were then centrifuged (1600 rpm for 90 s) to separate the plasma from the rest of the sample. After reacting the plasma samples (10 µL for each of the two tests) with the respective reagents for the d-ROMs test and PAT, the respective cuvettes were placed in the photometric analytical device (FRAS 5) [57].

#### 2.2.5. d-ROMs and PAT Measurements

The d-ROMs Fast test (for determining the concentration of peroxides) was used to measure reactive oxygen species. The plasma antioxidant test (PAT, measurement of iron-reducing capacity) was used to measure the antioxidant capacity. Both tests are part of the FRAS kit (Parma, IT) [57].

The measurement of the d-ROMs and the antioxidant capacity of plasma was carried out both in the peripheral blood sample (measurement of systemic OS) and in the blood sample taken from the penile corpora cavernosa (measurement of local OS).

The unit of measurement used in the d-ROMs test was Carratelli units (Carr. U.) [51,52]. The unit of measurement used in the PAT was Cornelli units (Cor. U.) [51,52]. For d-ROMs (d-ROMs test), the reference range of normal values is between 250 and 300 Carr. U. For the measurement of the antioxidant capacity of plasma (PAT), values between 2200 and 2800 Cor. U. are considered normal, while values < 1800 Cor. U. are considered deficient [51,52].

We calculated the OS index (OSI) using the following formula: OSI (arbitrary unit) = d-ROMs/PAT × 100 [51,52,53,54,55,56].

#### 2.2.6. Statistical Analysis

We used MedCalc statistical software (version 16.4.3, 2016) for the unpaired *t*-test and chi-square test. For the statistical study of the standard deviation, mean, median, and interquartile range calculation (IQR), we used CalculatorSoup^®^ software (version of 7 March 2023). SPSS Statistics software version 22.0 (2013) was used to calculate the Pearson correlation coefficient (size, strength, and direction of the relationship between two variables) and to perform the Mann–Whitney U-test (Wilcoxon rank sum) and Shapiro–Wilk test to test the normality of values. To explore the diagnostic ability of penile OSI and create a ROC curve, we used Eng J. ROC analysis: web-based calculator for ROC curves. Baltimore: Johns Hopkins University (version of 17 February 2022), link “http://www.rad.jhmi.edu/jeng/javarad/roc/JROCFITi.html (accessed on 31 October 2023)”. To calculate the optimal cut-off, related to the ROC curve (Youden index), for measuring the sensitivity and specificity of the test, we used the Youden index calculator (MDApp, (version of 29 June 2020, Manchester, UK). For the statistical study of logistic regression, we used AgriMetSoft’s software (2023 version) and Excel (MS Office, Redmond, WA, USA, 2011 version). In the statistical analyses, a threshold of 5% for alpha error (significant *p*-value < 0.05) was considered to demonstrate statistical significance.

## 3. Results

The demographic and clinical characteristics of the 49 PD patients and control group subjects are shown in Table 1 and Table 2.

No statistically significant differences were detected between the demographic and clinical characteristics of the group of PD patients and the control group subjects (see Table 2).

The values of the d-ROMs, PAT, and the relative OS index (both systemic and penile) relating to the PD patients and control group subjects are listed in Tables S1 and S2 in the Supplementary Materials.

**Figure 1:**
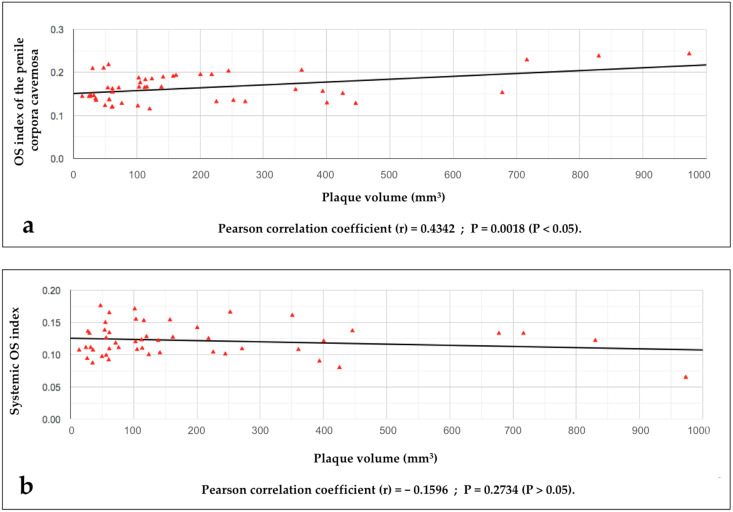
Graph highlighting the relationship between plaque volumes and penile OS index values (**a**) and between plaque volumes and systemic OS index values (**b**).

The Pearson correlation coefficient (PCC) statistical study demonstrated a statistical correlation between the penile OSI values and the volumes of the disease area (PD plaque): PCC = r 0.4342, r^2^ 0.1885, *p*-value = 0.001827 (*p* < 0.05) (see Figure 1).

The Pearson correlation coefficient statistical study did not demonstrate a statistical correlation between the systemic OSI values and the volumes of the disease area (plaque): PCC = r − 0.1596, r^2^ 0.02546, *p* = 0.2734 (*p* > 0.05) (see Figure 1).

The Pearson correlation coefficient statistical study did not demonstrate a statistical correlation between the systemic OSI values and penile OSI values: Pearson correlation coefficient, *p*-value = 0.198 (*p* > 0.05); furthermore, there was a statistically significant difference between the systemic OSI values and penile OSI values: unpaired *t*-test, *p*-value < 0.0001 (*p* < 0.05).

The comparison between the penile OSI values of the PD patients (before treatment) and the penile OSI values of the control group subjects revealed statistically significant differences (*p*-value < 0.0001), (*p* < 0.05).

We ascertained that the penile OSI values were significantly reduced after treatment and specifically after elimination of PD plaque (Mann–Whitney U-test, Z-score = 6.05369, *p*-value < 0.00001).

**Figure 2:**
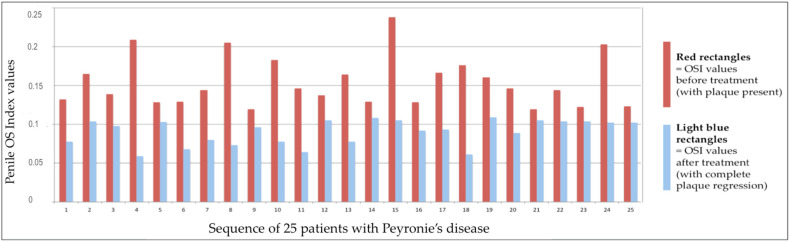
OSI values of penile corpora cavernosa before and after treatment and elimination of PD plaque.

Figure 2 highlights the decrease in the penile OSI values after the treatment and the disappearance of Peyronie’s plaque.

Our study allowed for us to identify in the PD patients (after plaque elimination) a mean value of penile OSI indices corresponding to 0.090 ± 0.016 (*p*-value = 0.004, *p* < 0.05). In the control group subjects, a mean value of penile OSI indices was obtained corresponding to 0.096 ± 0.016 (*p*-value = 0.006, *p* < 0.05).

The comparison between the penile OSI values of the PD patients with plaque elimination and the penile OSI values of the control group subjects revealed no statistically significant differences (*p*-value = 0.130, *p* < 0.05).

**Figure 3:**
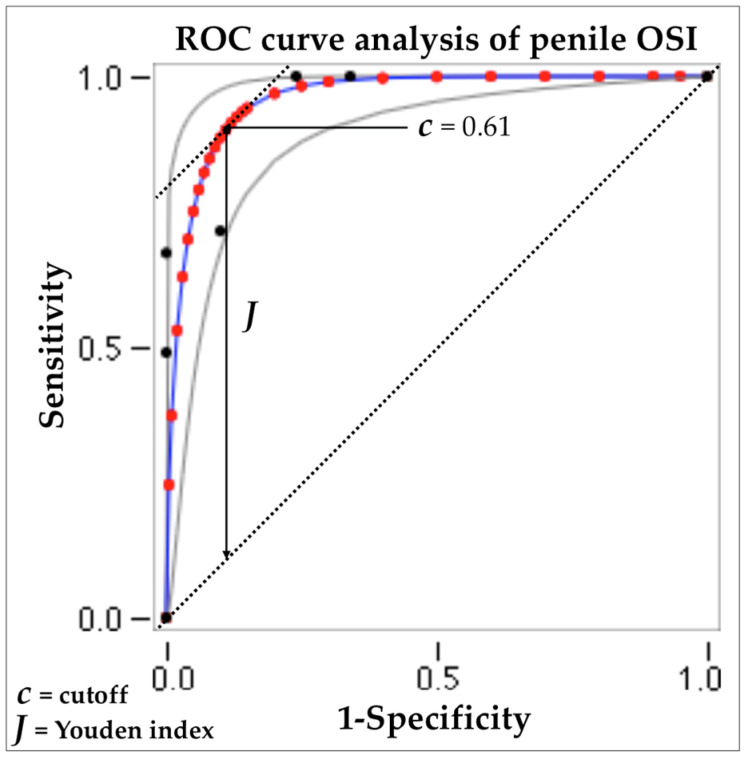
Analysis of the ROC curve of penile OSI and identification of the cutoff (Youden index).

In the ROC curve analysis, the area under the curve that represents the actual measure of accuracy was found to be 0.959, significance level *p*-value < 0.0001, sensitivity 71.4%, and specificity 90%; furthermore, after calculating the Youden index, it was found that the penile OSI value equal to 0.61 represented the optimal cutoff value (see Figure 3).

No correlation has been established between OSI values (both systemic and penile) and associated pathological conditions including cigarette smoking (see Table 3).

Table 4 illustrates our results regarding the prevalence of depression and anxiety in 49 PD patients.

## 4. Discussion

This is the first study where the OS index (OSI) was calculated by analyzing OS directly in the penile corpora cavernosa of PD patients by taking a blood sample.

To measure the antioxidant capacity, until some time ago, the biological antioxidant potential test (BAP test) was generally used, which is based on the ability of blood plasma to reduce the ferric ion to a ferrous ion. However, this method has an evaluation bias, as the colorimetric determination of the ferric ion is influenced by the phosphate ions. In practice, part of the ferric ions complexed with the thiocyanate ion binds to the phosphate ions normally present in the plasma sample, causing discoloration of the solution, which does not depend on the actual concentration of antioxidants but on the concentration of the phosphate ions. The result of this chemical reaction translates into a significant overestimation of the antioxidant capacity. With the more modern PAT, which is similarly based on the colorimetric determination of the ferric ion, a zirconium salt with adequate concentrations was introduced into the test, which is a transition metal that has a greater affinity with phosphates [52]. This made it possible to eliminate the interference caused by plasma phosphates.

The results of our study reinforce the observation that OS plays an important role in the pathogenesis of PD [15,16,17,18,19,20,21,22,23,70]. Penile OSI could be considered a biochemical parameter to diagnose the extent of PD given that OSI values were found to correlate closely with plaque volume.

Having detected a significant correlation between the volumes of Peyronie’s plaque and the penile OSI values, we could have access not only to the ultrasound study (which allows for us to detect the volume of Peyronie’s plaque) but also an important marker that could indicate the real presence of the disease, confirming the therapeutic result not only visually but also chemically.

Furthermore, in the absence of a certain ultrasound diagnosis, which is possible in some cases, the study of OS in the penile corpora cavernosa could prove to be a test for a more certain and definitive diagnosis.

The absence of a correlation between the volumes of Peyronie’s plaque and the systemic OSI indices indicates that, to be truly meaningful, the study of OS must be performed by taking a sample directly from the site of the disease, as has already been carried out in other studies for other pathologies [49,50,71].

Given that the comparison between the penile OSI values of the PD patients with plaque elimination and the penile OSI values of the control group subjects did not reveal statistically significant differences, it indicates that the study of penile OSI can represent an effective test for the study of oxidative markers in this disease.

Even if the aim of this study was not the following, our results testified to the elimination of the plaque after antioxidant treatment, as other articles in the literature have already described [72,73,74,75].

Even if this study did not demonstrate any correlation between penile OSI values and anxious–depressive state, we detected a high prevalence of anxiety (81.6%) and depression (59.1%) in the PD patients. These data confirm that the anxious–depressive state represents one of the symptoms of PD (along with penile pain, penile curvature/deformation, and ED) [10,76,77,78]. The finding of significant percentages of anxious–depressive disorders has also been noted by other authors who have used the Peyronie’s Disease Questionnaire (PDQ) domains [79]. Although our results are interesting, the limitation of our study was that we did not have a very large sample of patients.

Further studies are needed to investigate OS in the penile corpora cavernosa of subjects with PD. To optimize this clinical research, experimental studies on animal models could also be implemented. For example, experimental studies on rats would be necessary to induce penile fibrosis by injecting fibrogenic agents (TGF-β, fibrin, or others) into the penile tunica albuginea, as has already been conducted in other studies [80,81,82]. After the formation of fibrotic areas in the penis, oxidative stress could be measured in both the corpora cavernosa and peripheral blood of rats. Subsequently, antioxidants or antifibrotics (such as anthocyanin, platelet-rich plasma, or others) could be administered into the penile corpora cavernosa of rats to reduce fibrosis [81,82]. Once this effect was achieved, OSI could be measured as it was performed before the treatment.

## 5. Conclusions

The absence of a correlation between the PD plaque volumes and systemic OSI indices and, on the contrary, the existence of a significant correlation between the PD plaque volumes and the penile OSI values, indicate that the study of OS must be carried out by taking a sample directly from the site of the disease; other authors have demonstrated this in other fields of specialist medicine.

The penile OSI study associated with the ultrasound study, which allows for us to examine the plaque volume, would also provide a precious oxidative marker that could indicate the real chemical presence of the disease.

Although our data are very interesting, further studies are needed to investigate OS (with the calculation of the OS index) in the penile corpora cavernosa of PD patients.

## Figures and Tables

**Table 1:** Demographic characteristics and plaque volume of the 49 patients with Peyronie’s disease.

	All n. 49 PD Patients	n. 25 PD Patients with Plaque Elimination	n. 24 PD Patients with Partial Plaque Elimination	Statistical Analysis 25 PD p. versus 24 PD p. *p*-Value (*t*-Test)
Mean age (years) (SD)	49.65 (±11.01)	46.68 (±11.29)	50.66 (±10.85)	0.214 *
Plaque volume (mm^3^) (SD)	194.33 (±218.55)	152.64 (±192.30)	237.75 (±239.30)	0.175 *
**n. 25 PD patients** **with plaque elimination**			**n. 24 PD patients** **with partial plaque** **elimination**	
Patient no.	Plaque volume (mm^3^)		Plaque volume (mm^3^)	
1.	34.9		113.4	
2.	26.4		76.2	
3.	13.7		350.8	
4.	35.6		101.5	
5.	24.3		138.8	
6.	123.8		112.3	
7.	105.3		200.5	
8.	27.4		71.4	
9.	716.4		102.9	
10.	120.2		973.0	
11.	393.7		445.9	
12.	56.3		425.4	
13.	60.4		830.0	
14.	54.0		30.0	
15.	61.4		400.4	
16.	61.4		271.3	
17.	56.4		225.7	
18.	157.4		116.0	
19.	103.5		61.3	
20.	49.7		252.5	
21.	244.8		141.4	
22.	677.5		161.9	
23.	218.3		47.4	
24.	360.4		55.2	
25.	31.9		-	

NOTE: PD = Peyronie’s disease; SD = standard deviation; * = no statistically significant difference found.

**Table 2:** Demographic and clinical characteristics of PD patients and control group subjects.

	Group of PD Patients (n. 49) Mean Age 49.65 Years (SD ± 11.01)		Control Group (n. 50) Mean Age 49.94 Years (SD ± 11.42)		Statistical Analysis Group-PD versus Control Group *p*-Value (*t*-Test) 0.912
**Demographic** **characteristics**	N. patients (out 49) (%)		N. patients (out 50) (%)		***p*-value** (chi-square test)
**Race**					
Caucasian	49 (100)		50 (100)		1.000
**Age Range**	-		-		-
From 20 to 30 years	2 (4.08)		3 (6.0)		0.713
From 31 to 40 years	10 (20.4)		9 (18.0)		0.762
From 41 to 50 years	13 (26.53)		8 (16.0)		0.202
From 51 to 60 years	14 (28.57)		20 (40.0)		0.233
From 61 to 70 years	10 (20.4)		10 (20.0)		0.959
**Type of school education**	-		-		
elementary school	2 (4.08)		1 (2.0)		0.547
secondary school	38 (77.55)		39 (78.0)		0.957
university degree	9 (18.36)		10 (20.0)		0.837
**Clinical condition** **associated in PD patients**	N. patients (out 49)		N. patients (out 50)		**Statistical analysis** **Group-PD** **versus** **Control group** ***p*-value** (*t*-test)
Anxiety	40	Mean GAD-7 score = 16.4	42	Mean GAD-7 score = 16.2	0.806
Depression	29	Mean PHQ-9 score = 15.2	32	Mean PHQ-9 score = 14.4	0.383
Penile curvature	45	Average penile curvature angle (degrees) = 35.1°	0	Average penile curvature angle (degrees) = 0°	<0.0001
Penile pain	26	Mean VAS score = 4.6	0	Mean VAS score = 0	<0.0001
Erectile dysfunction	19	Mean IIEF score = 21.9	50	Mean IIEF score = 22.4	0.469
Cigarette smoking	16	Mean No. of cigarettes per day = 9.3	17	Mean No. of cigarettes per day = 8.76	0.808

NOTE: PD = Peyronie’s disease; SD = standard deviation; *p*-value significant when <0.05; GAD-7 = Generalized Anxiety Disorder-7 questionnaire; PHQ-9, = Patient Health Questionnaire-9; IIEF = International Index of Erectile Function; VAS = visual analogue scale.

**Table 3:** Associated clinical conditions in 49 patients with Peyronie’s disease and the study of their possible statistical correlation with the “OS index values of the penile corpora cavernosa” or “systemic OS index values”.

Clinical Condition Associated in PD Patients	N. Patients (out 49)	Type of Statistical Analysis Used and (*p*-Value)		Correlation with Penile OS Index Values (YES or NO)	Type of Statistical Analysis Used and (*p*-Value)		Correlation with Systemic OS Index Values (YES or NO)
Anxiety	40	*t*-test (*p* = 0.153)	Pearson correlation coefficient (*p* = 0.094)	NO	*t*-test (*p* = 0.281)	Pearson correlation coefficient (*p* = 0.524)	NO
Depression	29	*t*-test (*p* = 0.781)	Pearson correlation coefficient (*p* = 0.901)	NO	*t*-test (*p* = 0.184)	Pearson correlation coefficient (*p* = 0.171)	NO
Penile pain	26	*t*-test (*p* = 0.221)	Pearson correlation coefficient (*p* = 0.081)	NO	*t*-test (*p* = 0.676)	Pearson correlation coefficient (*p* = 0.834)	NO
Penile curvature	45	*t*-test (*p* = 0.920)	Pearson correlation coefficient (*p* = 0.704)	NO	*t*-test (*p* = 0.796)	Pearson correlation coefficient (*p* = 0.290)	NO
Erectile dysfunction	19	*t*-test (*p* = 0.753)	Pearson correlation coefficient (*p* = 0.774)	NO	*t*-test (*p* = 0.611)	Pearson correlation coefficient (*p* = 0.655)	NO
Cigarette smoking	16	*t*-test (*p* = 0.488)	Pearson correlation coefficient (*p* = 0.380)	NO	*t*-test (*p* = 0.564)	Pearson correlation coefficient (*p* = 0.381)	NO

**Table 4:** Prevalence of depression and anxiety in 49 PD patients.

PHQ-9 Score Range		No. Total Cases (%)
**No depression**	**0**	**0**
**Minimal or mild depression**	**1–9**	**20 (40.8)**
**Moderate–Severe depression**	**10–27**	**29 (59.1)**
- Moderate depression	10–14	16 (32.6)
- Moderately severe depression	15–19	11 (22.4)
- Severe depression	20–27	2 (4.08)
**TOTAL**		**49**
**GAD-7 score range**		**No. total cases (%)**
**No anxiety**	**0**	**0**
**Minimal or mild anxiety**	**1–9**	**6 (12.2)**
**Moderate–Severe anxiety**	**10–21**	**40 (81.6)**
- Moderate anxiety	10–14	20 (40.8)
- Severe anxiety	15–21	20 (40.8)
**TOTAL**		**49**

NOTE: PD = Peyronie’s disease; PHQ-9 = Patient Health Questionnaire-9; GAD-7 = Generalized Anxiety Disorder Questionnaire-7.

## Data Availability

The original contributions presented in the study are included in the article/Supplementary Materials, further inquiries can be directed to the corresponding author.

## Supplementary Materials

The following supporting information can be downloaded at: https://www.mdpi.com/article/10.3390/metabo14010055/s1, Table S1: Values of d-ROMs, PAT and relative OSI index (“systemic” and “in penile corpora cavernosa”) of the 49 patients with Peyronie’s disease, Table S2: Values of d-ROMs, PAT and relative OSI index (“systemic” and “in penile corpora cavernosa”) of the 50 normal cases (control group).
