# Supplementary material for: Measurement of Oxidative Stress Index (OSI) in Penile Corpora Cavernosa and Peripheral Blood of Peyronie’s Disease Patients: A Report of 49 Cases
Source: Metabolites. 2024 Jan 15;14(1):55. doi: 10.3390/metabo14010055 (PMC10821449; doi:10.3390/metabo14010055)
Supplement: Supplementary file 1 [file metabolites-14-00055-s001.zip › metabolites-2793526-supplementary.pdf]

Table S1. Values of d-ROMs, PAT and relative OSI index ("systemic" and "in penile corpora cavernosa") of the 49 patients with Peyronie's disease.

	**d-ROMs values** Carratelli units (Carr. U.) systemic plasma sample	**PAT values** Cornelli units (Cor. U.) systemic plasma sample	**OSI index** systemic plasma sample	**d-ROMs values** Carratelli units (Carr. U.) penile plasma sample	**PAT values** Cornelli units (Cor. U.) penile plasma sample	**OSI index** penile plasma sample
1.	360	4242	**0.087**	414	3125	**0.132**
2.	354	3778	**0.094**	570	2949	**0.193**
3.	352	3283	**0.107**	390	2358	**0.165**
4.	498	4619	**0.107**	569	2910	**0.195**
5.	428	3841	**0.111**	395	2825	**0.139**
6.	344	3410	**0.100**	447	2680	**0.166**
7.	441	4057	**0.108**	616	2934	**0.209**
8.	470	3545	**0.136**	352	2590	**0.135**
9.	339	3055	**0.110**	543	2368	**0.229**
10.	282	2532	**0.111**	702	2885	**0.243**
11.	502	3114	**0.161**	410	3183	**0.128**
12.	539	3152	**0.171**	401	3102	**0.129**
13.	424	3176	**0.133**	513	3557	**0.144**
14.	354	2756	**0.128**	605	2941	**0.205**
15.	390	4288	**0.090**	412	3562	**0.115**
16.	390	3080	**0.126**	535	2825	**0.189**
17.	321	2631	**0.122**	439	2389	**0.183**
18.	291	3157	**0.092**	438	2665	**0.164**
19.	358	2576	**0.138**	508	2716	**0.187**
20.	352	2842	**0.123**	464	2964	**0.156**
21.	580	4080	**0.142**	560	2564	**0.218**
22.	342	2885	**0.118**	416	2840	**0.146**
23.	392	3245	**0.120**	503	2631	**0.191**
24.	414	2643	**0.165**	403	2798	**0.144**
25.	246	1826	**0.134**	464	2787	**0.166**
26.	205	3123	**0.065**	479	2941	**0.162**
27.	280	2821	**0.099**	382	2885	**0.132**
28.	418	3042	**0.137**	415	2737	**0.151**
29.	438	2830	**0.154**	436	3216	**0.135**
30.	485	3162	**0.155**	399	2892	**0.137**
31.	245	3052	**0.080**	400	2430	**0.164**
32.	324	2641	**0.122**	448	3719	**0.120**
33.	314	3218	**0.097**	725	3045	**0.238**
34.	341	3353	**0.101**	506	3936	**0.128**
35.	422	3157	**0.133**	399	2928	**0.136**
36.	326	2641	**0.121**	572	3440	**0.166**
37.	339	3096	**0.109**	498	2823	**0.176**
38.	300	2861	**0.104**	551	3442	**0.160**
39.	603	3937	**0.153**	521	3564	**0.146**
40.	387	3526	**0.109**	555	2991	**0.185**
41.	498	2990	**0.166**	534	2537	**0.210**
42.	379	2842	**0.133**	612	3985	**0.153**
43.	338	2685	**0.125**	410	2652	**0.154**
44.	330	2034	**0.108**	425	3564	**0.119**
45.	319	3080	**0.103**	409	2840	**0.144**
46.	359	2806	**0.127**	563	4608	**0.122**
47.	435	2464	**0.176**	525	2685	**0.195**
48.	468	4199	**0.111**	538	2641	**0.203**
49.	538	3576	**0.150**	384	3111	**0.123**

NOTE : d-ROMs = derived from the Reactive Oxygen Metabolites; PAT = Plasma Antioxidant Test; OSI = Oxidative Stress Index.

Table S2. Values of d-ROMs, PAT and relative OSI index ("systemic" and "in penile corpora cavernosa") of the 50 normal cases (control group).

	**d-ROMs values** Carratelli units (Carr. U.) systemic plasma sample	**PAT values** Cornelli units (Cor. U.) systemic plasma sample	**OSI index** systemic plasma sample	**d-ROMs values** Carratelli units (Carr. U.) penile plasma sample	**PAT values** Cornelli units (Cor. U.) penile plasma sample	**OSI index** penile plasma sample
1.	434	3920	**0.110**	356	3465	**0.102**
2.	443	4057	**0.109**	309	3704	**0.080**
3.	476	4085	**0.116**	348	2939	**0.118**
4.	392	3516	**0.111**	307	2956	**0.103**
5.	385	3843	**0.100**	169	3836	**0.044**
6.	315	2951	**0.106**	294	3327	**0.088**
7.	539	4461	**0.120**	309	3320	**0.093**
8.	373	3651	**0.102**	387	3412	**0.084**
9.	269	2763	**0.097**	299	3217	**0.092**
10.	295	3419	**0.093**	242	3316	**0.072**
11.	329	3544	**0.092**	258	3017	**0.085**
12.	334	3034	**0.110**	219	3302	**0.066**
13.	289	2842	**0.101**	258	3064	**0.084**
14.	323	2756	**0.117**	288	2998	**0.096**
15.	423	3632	**0.116**	315	2895	**0.108**
16.	425	3544	**0.119**	350	2971	**0.117**
17.	415	3622	**0.114**	261	2891	**0.092**
18.	334	4006	**0.083**	222	2955	**0.075**
19.	299	2984	**0.100**	262	3103	**0.084**
20.	403	3719	**0.103**	288	2821	**0.102**
21.	377	3848	**0.097**	250	2860	**0.087**
22.	441	4613	**0.095**	279	3005	**0.092**
23.	372	3914	**0.095**	312	3416	**0.091**
24.	322	3735	**0.086**	305	4101	**0.074**
25.	275	3283	**0.083**	238	3572	**0.092**
26.	344	3739	**0.092**	313	2981	**0.104**
27.	272	4315	**0.063**	300	3704	**0.080**
28.	382	4461	**0.085**	411	4057	**0.101**
29.	367	4901	**0.074**	437	4324	**0.101**
30.	341	4461	**0.076**	386	5426	**0.071**
31.	401	3824	**0.104**	415	3946	**0.105**
32.	409	4652	**0.087**	325	3112	**0.104**
33.	410	3753	**0.109**	377	3352	**0.112**
34.	499	4489	**0.111**	451	3854	**0.117**
35.	371	3224	**0.114**	321	3252	**0.098**
36.	513	4922	**0.104**	361	3955	**0.091**
37.	294	2785	**0.105**	261	2551	**0.102**
38.	448	4370	**0.102**	410	3891	**0.103**
39.	363	3712	**0.097**	341	3096	**0.110**
40.	371	3597	**0.103**	372	3263	**0.114**
41.	358	3515	**0.101**	412	3597	**0.114**
42.	318	3055	**0.104**	352	2992	**0.117**
43.	511	4947	**0.103**	485	4295	**0.112**
44.	436	4126	**0.105**	452	3896	**0.116**
45.	401	4075	**0.098**	428	3877	**0.110**
46.	519	4341	**0.119**	536	4871	**0.110**
47.	430	4080	**0.105**	417	3597	**0.115**
48.	482	4540	**0.106**	431	3690	**0.116**
49.	288	2550	**0.112**	251	2116	**0.118**
50.	402	4134	**0.097**	286	4387	**0.065**

NOTE : d-ROMs = derived from the Reactive Oxygen Metabolites; PAT = Plasma Antioxidant Test; OSI = Oxidative Stress Index.
